# NP108, an Antimicrobial Polymer with Activity against Methicillin- and Mupirocin-Resistant Staphylococcus aureus

**DOI:** 10.1128/AAC.00502-17

**Published:** 2017-08-24

**Authors:** Derry K. Mercer, Laura K. Katvars, Fiona Hewitt, Daniel W. Smith, Jennifer Robertson, Deborah A. O'Neil

**Affiliations:** NovaBiotics Ltd., Craibstone, Aberdeen, United Kingdom

**Keywords:** antimicrobial activity, nasal decolonization, Staphylococcus aureus

## Abstract

Staphylococcus aureus is a clinically significant human pathogen that causes infectious diseases ranging from skin and soft tissue infections (SSTI) and health care-associated infections (HAI) to potentially fatal bacteremia and endocarditis. Nasal carriage of S. aureus, especially for persistent carriage, is associated with an increased risk of subsequent infection, particularly nosocomial and surgical site infections (SSI), usually via autoinfection. NP108 is a cationic antimicrobial polymer composed of generally recognized as safe (GRAS) amino acid building blocks. NP108 is broad spectrum and rapidly bactericidal (3-log kill in ≤3 h), killing bacteria by membrane disruption and cell lysis. NP108, contrary to many antibiotics, shows equally effective antimicrobial activity against a variety of S. aureus (MIC_100_ = 8 to 500 mg/liter) and S. epidermidis (MIC_100_ = 4 to 8 mg/liter) isolates, whether exponentially growing or in stationary phase. NP108 is antimicrobially active under nutrient-limiting conditions similar to those found in the anterior nares (MIC_100_ = 8 mg/liter) and kills antibiotic-resilient small colony variants (MIC_100_ = 32 mg/liter) and S. aureus biofilms (prevention, MIC_100_ = 1 to 4 mg/liter; eradication, MIC_100_ ≥ 31.25 mg/liter). NP108 is active against isolates of S. aureus resistant to the current standard-of-care decolonization agent, mupirocin, with no significant increase in the MIC_100_. NP108 is water soluble and has been formulated into compatible aqueous gel vehicles for human use in which antimicrobial efficacy is retained (2.0% [wt/vol]). NP108 is a potential nonantibiotic antimicrobial alternative to antibiotics for the nasal decolonization of S. aureus, with clear advantages in its mechanism of action over the existing gold standard, mupirocin.

## INTRODUCTION

Staphylococcus aureus is a major human pathogen, despite existing as a commensal colonizer of the skin in ∼30% of the global population (1–3). S. aureus is a leading cause of bacterial health care-associated infections (HAI), including surgical site infections (SSI), pleuropulmonary infections, skin and soft tissue infections (SSTI), and osteomyelitis and device-related infections, as well as potentially fatal infections, including bacteremia, endocarditis, and toxic shock syndrome (1–3). Since the advent of the antibiotic era, S. aureus has demonstrated a remarkable ability to adapt and develop resistance to almost all antibiotic classes. Methicillin-resistant Staphylococcus aureus (MRSA) is resistant to all available penicillins and other β-lactams (4) and, in some cases, other antibiotic classes, including macrolides and tetracyclines (5). Data for 2015 indicate that 10 to 12% of invasive S. aureus isolates in the United Kingdom were MRSA (6), and there were an estimated 59,000 cases of MRSA infection and 9,670 attributable deaths in the United States in 2012 (7).

Approximately one-quarter to one-third of humans are asymptomatic persistent nasal carriers of S. aureus, whereas a further ∼30% are intermittent carriers (8, 9). Nasal carriage of S. aureus, especially for persistent carriage, has been positively correlated to an increased risk of subsequent infection, usually via autoinfection (10–12). The World Health Organization recently published 29 (13 preoperative and 16 intra- and postoperative) global recommendations for the prevention of SSI, including nasal decolonization with mupirocin ointment in patients with known nasal carriage of S. aureus (13, 14). The frequent overuse of mupirocin has quickly been followed by mupirocin resistance (low and high level) with some outbreaks of mupirocin-resistant MRSA reported (15, 16). Mupirocin resistance rates as high as 81% have been reported and are significantly associated with persistent MRSA carriage, decolonization failure, and early recolonization (17–21).

The emergence of mupirocin resistance and examples of decolonization failure means that alternative S. aureus decolonization strategies are actively being sought for both mupirocin nasal decolonization and chlorhexidine gluconate washing (16, 22). The use of the lysine polymer, NP108, represents one such alternative.

Antimicrobial peptides (AMPs) are key components of the innate immune system, with examples of AMPs found in all taxonomic kingdoms (23–26). Most endogenous AMPs are cationic (due to the net charge effect of the arginine and lysine content), amphipathic, contain 10 to 50 amino acids, and target the anionic microbial cell membrane, resulting in cell destabilization, membrane lysis, and death (27–30). Polylysines (modified epsilon forms) have been used as preservatives for a range of dairy and meat products (31, 32). With this precedent for synthetic/modified polylysines and natural, lysine-rich AMPs, we therefore investigated whether simple polymers of natural lysines were active against antibiotic-sensitive and antibiotic-resistant S. aureus, including methicillin-susceptible Staphylococcus aureus (MSSA), MRSA, and mupirocin-sensitive and -resistant isolates. In this manuscript, we describe the *in vitro* activity of NP108, a lysine polymer selected for development for the nasal decolonization of S. aureus (MSSA and MRSA) and the prevention of HAI. NP108 is a broad-spectrum rapidly acting bactericidal compound that kills bacterial cells by membrane disruption and cell lysis through mechanisms aligned with those described for endogenous lysine-rich AMPs (31). Our data also demonstrate that NP108, as a highly water-soluble polymer, can be formulated into compatible delivery vehicles for use in humans (application to the anterior nares) without a loss of antimicrobial efficacy.

## RESULTS AND DISCUSSION

### Antimicrobial activity of NP108 versus S. aureus and S. epidermidis.

The antimicrobial efficacy of NP108 against a panel of 28 S. aureus (8 MRSA and 20 MSSA) isolates and 3 S. epidermidis isolates was determined by the broth microdilution procedure (see Table S1 in the supplemental material) (33). NP108 MIC_100_ values for all but two of the isolates tested were in the low milligram per liter range (4 to 64 mg/liter), and for the two less-NP108-sensitive isolates, higher MICs (500 mg/liter for S. aureus CB005 and 250 mg/liter for S. aureus CB007) were recorded and compared favorably with those of epsilon-polylysine and other AMPs (31). The minimum bactericidal concentrations (MBCs) of NP108 against these Staphylococcus species isolates were determined and all were identical to the MIC_100_ or within one dilution of the MIC_100_, indicating that NP108 is bactericidal after 20 h, unlike mupirocin, which is bacteriostatic as MBC values (mg/liter) are significantly higher than MIC values (Table 1) (34).

**TABLE 1:** Antimicrobial activity of mupirocin against Staphylococcus spp^*a*^

Isolate	Features	Mean MIC_100_ (mg/liter)	MBC (mg/liter)
S. aureus DSMZ11729	MRSA	0.25	>4.0
S. aureus NCTC10442	MRSA	0.50	>8.0
S. aureus BAA-1717	MRSA	0.50	>8.0
S. aureus ATCC 25923	MSSA	0.25	>8.0
S. aureus NS05R1	MSSA, nasal isolate	1.00	8.0
S. aureus NCTC10788	MSSA	0.25	>4.0
S. aureus NCTC6571	MSSA	0.25	>4.0
S. epidermidis ATCC 12228	MSSE	0.25	>8.0
S. epidermidis V1A	MSSE, nasal isolate	0.50	>8.0

a The MIC at which 100% growth was inhibited (MIC_100_) and the minimum bactericidal concentration (MBC) at which all cells were killed by mupirocin were determined by the broth microdilution procedure (55) and as described in reference 33, respectively. Results represent the means from 3 experiments which each contained three technical replicates. MSSA, methicillin-sensitive S. aureus; MRSA, methicillin-resistant S. aureus; MSSE, methicillin-sensitive S. epidermidis.

### *In vitro* antimicrobial activity of NP108 against low-level mupirocin-resistant mutants of S. aureus.

Mupirocin is a competitive inhibitor of bacterial isoleucyl-tRNA synthetase, thereby blocking bacterial protein and RNA synthesis (16, 35). Mupirocin resistance can be categorized as low level (8 to 256 mg/liter) or high level (≥512 mg/liter), and isolates of MSSA and MRSA with both resistance types have been described (36–39). Low-level mupirocin-resistant mutants were generated from two strains of MSSA (S. aureus NCTC10788 and S. aureus NS05R1) and two strains of MRSA (S. aureus DSMZ11729 and S. aureus NCTC10442). Antibiotic susceptibility testing (Table 2) demonstrated that the mutants were resistant to mupirocin (MIC_100_ = 8 to 16 mg/liter), unlike the isogenic mupirocin-sensitive isolates (MIC_100_ < 1 mg/liter). In all cases, the mupirocin-resistant mutants remained equally sensitive to NP108, and in two cases (S. aureus DSMZ11729 and S. aureus NS05R1), the NP108 MIC was halved. The NP108 MIC values between isogenic mupirocin-resistant and mupirocin-sensitive strains were not statistically significant (unpaired *t* test [GraphPad Prism 4 for Windows]). This demonstrates that there is no evidence of cross-resistance between low-level mupirocin resistance and NP108 efficacy.

**TABLE 2:** Antimicrobial activity of NP108 and mupirocin against S. aureus and isogenic low-level mupirocin-resistant mutants^*a*^

Isolate	Mean MIC_100_ (mg/liter)	
	NP108	Mupirocin^*b*^
S. aureus DSMZ11729 (MRSA)	32	0.5 (S)
S. aureus DSMZ11729 (MRSA) Mup^r^	16	8 (R)
S. aureus NCTC10442 (MRSA)	32	<1 (S)
S. aureus NCTC10442 (MRSA) Mup^r^	32	16 (R)
S. aureus NCTC10788 (MSSA)	8	0.25 (S)
S. aureus NCTC10788 (MSSA) Mup^r^	8	>8 (R)
S. aureus NS05R1 (MSSA)	16	<1 (S)
S. aureus NS05R1 (MSSA) Mup^r^	8	16 (R)

a The MIC at which 100% growth was inhibited (MIC_100_) was determined by the broth microdilution procedure (33). Results represent the means from 3 experiments which each contained three technical replicates. Mupirocin susceptibility, MIC ≤ 4 mg/liter; low-level mupirocin resistance, MIC = 8 to 64 mg/liter; high-level mupirocin resistance, MIC ≥ 512 mg/liter (35); MSSA, methicillin-sensitive S. aureus; MRSA, methicillin-resistant S. aureus; Mup^r^, mupirocin resistant.

b R, resistant; S, sensitive.

### Antimicrobial activity of NP108 against small-colony variants of S. aureus.

Small-colony variants (SCV) of S. aureus are known to have reduced membrane potential (ΔΨ) compared with that the wild-type (WT) parental strain (40), and this has been shown to adversely affect aminoglycoside (e.g., gentamicin and tobramycin) antibacterial efficacy, as ΔΨ facilitates the uptake of aminoglycoside into bacterial cells (41). A reduced ΔΨ is also known to adversely affect the efficacy of selected cationic antimicrobial peptides (42–44), and as NP108 is cationic, it was deemed relevant to determine whether the susceptibility of S. aureus SCV to NP108 was affected. In a previous study that examined the susceptibility of S. aureus WT and SCV to cationic peptides, SCV were less susceptible to protamine and equally susceptible to magainin and human neutrophil peptide-1, but were more susceptible to dermaseptin (45). S. aureus SCV have been isolated from sinonasal biopsy specimens of chronic rhinosinusitis patients (46) and can be internalized by and maintained in a human keratinocyte (HaCaT) cell line *in vitro* (47). Therefore, SCV may be associated with S. aureus carriage in the anterior nares. SCV of one strain of MRSA (S. aureus DSMZ11729) and one of MSSA (S. aureus ATCC 25923) were generated, and their susceptibilities to NP108, mupirocin, and tobramycin were determined (Table 3). The MIC_100_ values of NP108 against S. aureus ATCC 25923 WT and SCV were identical (32 mg/liter), whereas the MIC_100_ of S. aureus DSMZ11729 SCV was one dilution higher than the MIC_100_ of the WT (64 and 32 mg/liter, respectively) (Table 2), indicating that SCV status has little or no effect on NP108 antibacterial efficacy against these isolates. The MICs of mupirocin were identical for both strains of S. aureus, whether WT or SCV (0.5 mg/liter). As expected, the susceptibilities of the SCV to the aminoglycoside antibiotic tobramycin were reduced, with >16-fold increases in MICs for S. aureus DSMZ11729 (tobramycin resistant and known to be gentamicin resistant [>50 mg/liter]) (48) and S. aureus ATCC 25923 SCV (Table 3). The data were analyzed by one-way analysis of variance (ANOVA), and the differences in antimicrobial susceptibility between the WT and SCV were not statistically significant (*P* > 0.05). Interestingly, S. aureus ATCC 25923 WT was tobramycin sensitive (MIC_100_ = 0.5 mg/liter), whereas S. aureus ATCC 25923 SCV was of intermediate sensitivity to tobramycin and potentially resistant (MIC_100_ > 8 mg/liter). Despite the fact that S. aureus DSMZ11729 was tobramycin resistant (MIC_100_ = 16 mg/liter), there was still an increase in tobramycin MIC (>16-fold) in the SCV isolate (MIC_100_ > 256 mg/liter).

**TABLE 3:** Antimicrobial activity of NP108, mupirocin, and tobramycin against wild-type and SCV of S. aureus^*a*^

Isolate	Mean MIC_100_ (mg/liter)		
	NP108	Tobramycin	Mupirocin
S. aureus DSMZ11729 (MRSA) WT	32	16	0.5
S. aureus DSMZ11729 (MRSA) SCV	64	>256	0.5
S. aureus ATCC25923 (MSSA) WT	32	0.5	0.5
S. aureus ATCC25923 (MSSA) SCV	32	>8	0.5

a The MIC at which 100% growth was inhibited (MIC_100_) was determined by the broth microdilution procedure (33). Results represent the means from 3 experiments which each contained three technical replicates. SCV, small-colony variant; WT, wild type. One-way ANOVA was conducted using GraphPad Prism 4 for Windows.

### Antimicrobial activity of NP108 versus S. aureus under low-nutrient conditions.

The anterior nares are a relatively nutrient-poor environment (49); therefore, for any antimicrobial intended for application for nasal decolonization, it is essential that antimicrobial activity is not compromised under nutrient-limiting conditions in which bacteria are not multiplying or are multiplying slowly. Many antibiotics are not as efficacious under these conditions (50) and therefore may not be suitable for nasal decolonization; but, membrane-active compounds may not be subject to such limitations (51). NP108 was more effective under low-nutrient conditions than under high-nutrient conditions, with the MIC_100_ reduced by 3-fold under low-nutrient conditions for both strains of S. aureus (Table 4). Antimicrobial susceptibility differences under low- and high-nutrient conditions were analyzed by one-way ANOVA. The differences between NP108 MICs under low- and high-nutrient conditions were statistically significant (*P* < 0.001), whereas they were not statistically significant for mupirocin (*P* > 0.05). Mupirocin was not adversely affected by low-nutrient conditions, with the MIC_100_ reduced by 4-fold in the case of S. aureus DSMZ11729 and being unaffected in the case of S. aureus ATCC 25923.

**TABLE 4:** Antimicrobial activity of NP108 and mupirocin against S. aureus DSMZ11729 and S. aureus ATCC 25923 under high- and low-nutrient conditions^*a*^

Isolate	Mean MIC_100_ (mg/liter)			
	NP108		Mupirocin	
	High nutrient	Low nutrient	High nutrient	Low nutrient
S. aureus DSMZ11729	32	8	0.5	0.125
S. aureus ATCC 25923	32	8	0.5	0.5

a Antimicrobial susceptibility testing was conducted by the broth microdilution procedure (33). High-nutrient condition testing was carried out in 1× CA-MH broth and low-nutrient condition testing was carried out in 0.1× CA-MH broth. Results represent the means from 3 experiments which each contained three technical replicates. One-way ANOVA was conducted using GraphPad Prism 4 for Windows.

### Kinetics of NP108 killing of exponential- and stationary-phase S. aureus.

**FIG 1:**
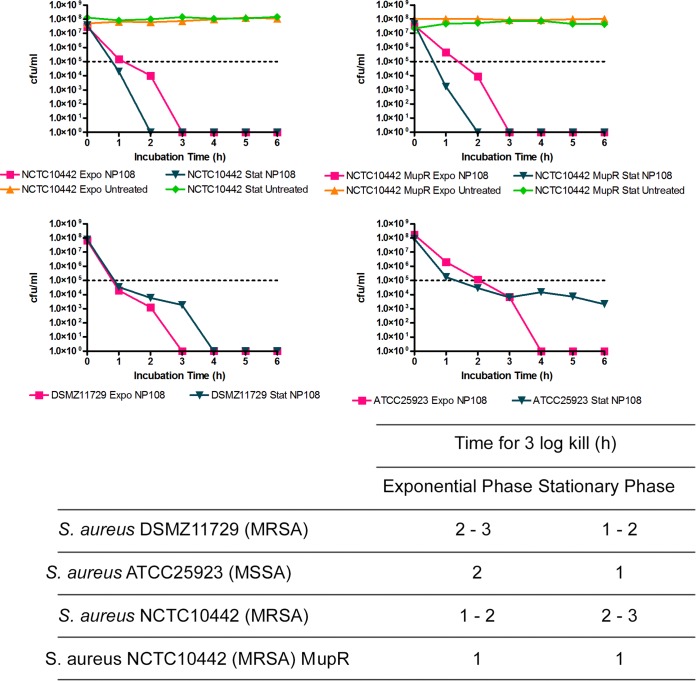
Effect of NP108 on the survival of S. aureus DSMZ11729 (MRSA), S. aureus ATCC 25923 (MSSA), S. aureus NCTC10442 (MRSA), and S. aureus NCTC10442 Mup^r^ (low-level mupirocin-resistant MRSA) over 6 h of incubation. NP108 was added at 4× MIC to exponential-phase or stationary-phase cultures of the S. aureus isolates diluted to the 0.5 McFarland standard (∼10^8^ CFU/ml) at 37°C in CA-MH broth (exponential phase) or conditioned CA-MH broth (stationary phase). Samples were taken every 60 min and viable counts established by plating on CA-MH agar and incubating for 24 h at 37°C. Untreated cultures were used as growth controls. Values in the table represent the time taken to kill the isolates of three replicates from 3 independent experiments. The graphs show representative data from a single experiment with each isolate. Data points represent the mean numbers of CFU/ml within the experiment. Horizontal dotted lines represent 3-log reduction in CFU/ml (complete kill). Conditioned CA-MH broth was medium derived from cultures of the relevant S. aureus isolate grown to stationary phase (48 to 54 h), and cells were removed by centrifugation (5 min at 17,000 × *g*) and filter sterilized (0.22-μm PES filter).

Most bactericidal antimicrobials require bacteria that are actively growing/metabolizing to produce killing, whereas stationary-phase cultures of S. aureus demonstrate significant tolerance to many antibiotics (52, 53). Therefore, the antimicrobial activity of NP108 against exponential-phase and stationary-phase S. aureus DSMZ11729, S. aureus ATCC 25923, S. aureus NCTC10442 mupirocin-sensitive (Mup^s^), and S. aureus NCTC10442 mupirocin-resistant (Mup^r^) isolates was determined. The kinetics of NP108 (4× MIC) for killing 4 isolates of S. aureus were such that a 3-log kill was achieved within 3 h of incubation with an inoculum of ∼10^8^ CFU/ml of cells (Fig. 1). Stationary-phase cultures were killed as quickly as exponential-phase cultures. When the isogenic mupirocin-resistant isolate of S. aureus NCTC10442 was tested, it was killed more rapidly than the mupirocin-sensitive isolate, demonstrating that there was no evidence of cross-resistance with mupirocin and NP108. The 10^8^ CFU/ml represents ∼150-fold more cells than are used in broth microdilution antimicrobial susceptibility testing (33) but with only 4-fold more NP108 used. When 4× MBC mupirocin was used against 3 of the isolates (S. aureus DSMZ11729, S. aureus ATCC 25923, and S. aureus NCTC10442), no killing was achieved even after 24 h of incubation (data not shown), which correlates with the slow killing time of mupirocin even at high concentrations (34). It was not possible to test S. aureus NCTC10442 Mup^r^ with 4× MBC of mupirocin, as the mupirocin MBC (>1,024 mg/liter) of this isolate could not be determined due to limits of the aqueous solubility of mupirocin.

### Importance of cationicity for the bactericidal mechanism of action of NP108.

**FIG 2:**
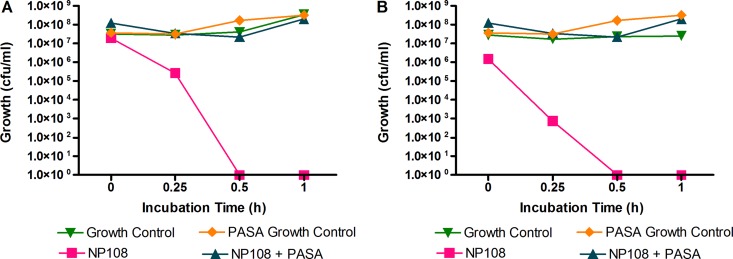
The effect of PASA on the antibacterial activity of NP108 against S. aureus MRSA. The influence of 2% (wt/vol) PASA in MH broth on the antibacterial efficacy of 62.5 mg/liter NP108 when incubated with S. aureus SMRSA105 (A) or S. aureus EMRSA16 (B) for 60 min at 37°C (*n* = 9). Data depict the mean numbers of CFU/ml.

NP108 is a cationic polylysine polymer (molecular weight [MW], ∼22), and the net positive charge is thought to be essential for the membrane-acting bactericidal activity of the compound. To test this hypothesis, the polyanionic compound polyanetholesulfonic acid (PASA) was included in the MIC assays along with NP108 and the antimicrobial efficacy was determined against S. aureus SMRSA105 and S. aureus EMRSA16 (Fig. 2). The addition of PASA to NP108 eliminated the antibacterial activity of NP108, while PASA had no effect on S. aureus survival alone. In the absence of PASA, NP108 rapidly killed both strains of S. aureus, indicating that the cationic properties of NP108 are essential for antimicrobial activity.

### Electron microscopy to determine the effect of NP108 on the cellular morphology of S. aureus.

**FIG 3:**
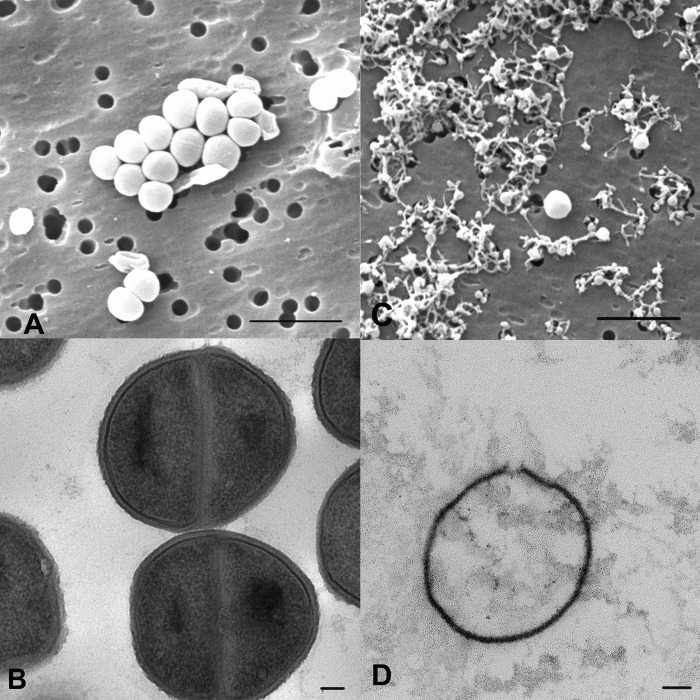
Electron micrographs demonstrating the effect of NP108 on S. aureus SMRSA105. (A) SEM, untreated; (B) TEM, untreated; (C) SEM, 62.5 mg/liter NP108 for 60 min; (D) TEM, 62.5 mg/liter NP108 for 60 min. Bars = 200 nm (A and C) and 100 nm (B and D).

NP108 has charge-dependent potent bactericidal effects within 1 h, as demonstrated in Fig. 2. Scanning and transmission electron microscopy (SEM and TEM, respectively) were used to investigate the effect of NP108 on the morphology of S. aureus SMRSA105. SEM analysis of a 60-min treatment with 60 mg/liter NP108 revealed extensive cellular debris with few intact cells (Fig. 3C). The TEM micrographs (Fig. 3D) revealed that, despite appearing intact, the remaining cells following incubation with 62.5 mg/liter NP108 no longer contained any internal architecture, unlike in the untreated control (Fig. 3B). There is also evidence of buckling and bulging of the cell walls in the treated samples, indicative of a loss of cell integrity and lysis, confirming the rapid bactericidal action of NP108 targeting the cell surface.

### Activity of NP108 against S. aureus biofilms.

**FIG 4:**
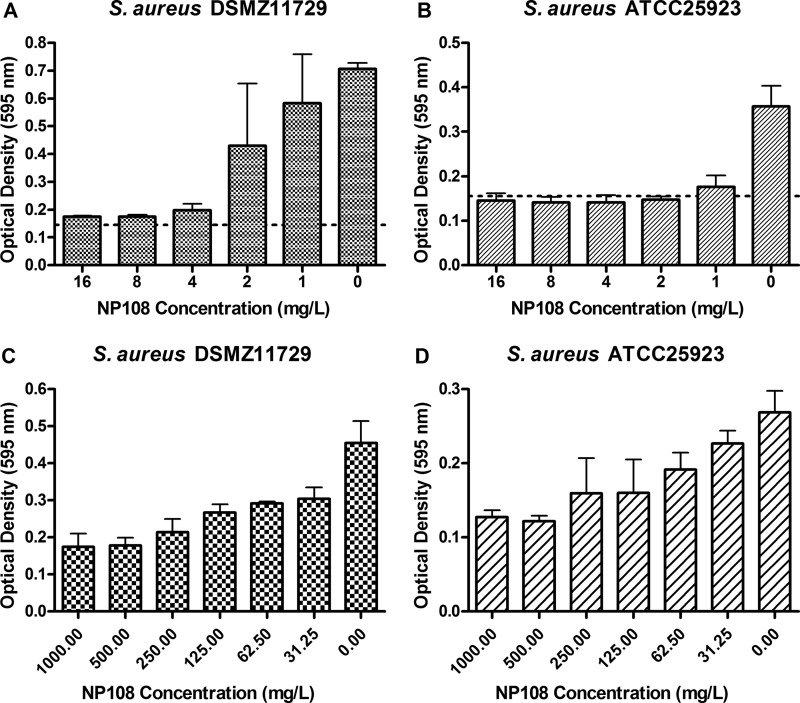
Effect of NP108 on prevention of biofilm formation by S. aureus DSMZ11729 (A) and S. aureus ATCC 25923 (B) under low-nutrient conditions simulating the anterior nares. Exponentially growing S. aureus isolates were diluted to the 0.5 McFarland standard in 0.1× TSB supplemented with 0.05% (wt/vol) glucose, 0.3% (wt/vol) NaCl, and 0 to 16 mg/liter NP108. Biofilms were allowed to establish statically for 96 h at 37°C before biomass formation was assessed by the crystal violet method. Data sets represent the means from triplicate experiments and error bars represent the standard errors of the means. Dotted lines represent the limit of detection. Effect of NP108 on established biofilms of S. aureus DSMZ11729 (C) and S. aureus ATCC 25923 (D) under low-nutrient conditions simulating the anterior nares. S. aureus biofilms were prepared in 0.1× TSB supplemented with 0.05% (wt/vol) glucose and 0.3% (wt/vol) NaCl and allowed to establish statically for 96 h at 37°C. Subsequently, biofilms were treated with different concentrations of NP108 in 0.1× TSB supplemented with 0.05% (wt/vol) glucose and 0.3% (wt/vol) NaCl for a further 24 h at 37°C before biofilm biomass eradication was assessed by the crystal violet method. Data points represent the mean optical densities (at 595 nm) from single experiments and error bars represent the standard errors of the means.

The antibiofilm activity of NP108 against established biofilms of S. aureus ATCC 25923 (MSSA) and S. aureus DSMZ11729 (MRSA) and whether NP108 could prevent biofilm formation by either strain were assessed (Fig. 4). NP108 disrupted S. aureus biofilms in a concentration-dependent manner from 31.25 to 500 mg/liter. Interestingly, biofilm prevention was achieved at lower concentrations (4 to 8 mg/liter) for both strains, which are concentrations lower than the MIC_100_ of NP108 against planktonic cells of either strain (32 mg/liter) (Table S1).

### Antimicrobial activity of NP108 prepared as water-soluble formulations.

NP108 has a high aqueous solubility (>400 g/liter), and so to generate a pharmaceutical formulation suitable for the application of NP108 to the anterior nares, the development of an aqueous formulation containing NP108 was a logical choice. This choice of formulation was based on the characteristics required for nasal application, including the retention of antimicrobial activity, the release of the antimicrobial from the formulation, the ease of application, appropriate hydrophilicity, a lack of irritation, and appropriate viscosity/rheological properties. NP108 retained antimicrobial activity against S. aureus ATCC 25923 (see Fig. S1B, D, F, H, and J) and other S. aureus isolates (data not shown), whereas none of the base formulations not containing NP108 demonstrated any antimicrobial activity (Fig. S1A, C, E, G, and I). Mupirocin (Bactroban nasal ointment) was also active under the same experimental conditions (Fig. S1K). Thus, NP108 can be included in suitable pharmaceutical formulations for use in subsequent human studies.

Our results from these studies describe the *in vitro* activity and development thus far of NP108, a novel antimicrobial lysine polymer composed of generally recognized as safe (GRAS) amino acid building blocks, for the nasal decolonization of S. aureus (MSSA and MRSA) and prevention of HAI. NP108 is rapidly bactericidal (99.9% kill within 3 h of exposure at 4× MIC against ∼10^8^ CFU/ml S. aureus) in the mg/liter concentration range against S. aureus and S. epidermidis and acts via charge-dependent membrane disruption, resulting in rapid cell lysis. NP108 shows *in vitro* activity against a variety of S. aureus and S. epidermidis strains, including nasal isolates, and is equally effective against exponentially growing or stationary-phase S. aureus and under nutrient-poor conditions similar to those found in the anterior nares (49). NP108 is effective against antibiotic-resilient small colony variants (SCV) and S. aureus biofilms. NP108 is also effective against mupirocin-resistant isolates of S. aureus. NP108 is water soluble and has been formulated into compatible vehicles for use in humans without a loss of antimicrobial efficacy. Subsequent studies will evaluate the *in vivo* efficacy of NP108 for S. aureus nasal decolonization. There is a clear need for a viable and superior alternative to mupirocin due to the increasing incidence of resistance to/treatment failure by this antibiotic. For example, NP108 has the potential to be superior due to its rapid bactericidal activity, which should significantly reduce the potential for resistance development compared with that of the existing relatively slow-acting bacteriostatic agent mupirocin, the current “gold standard” therapy. In addition, the data presented here show additional advantages for the use of a lysine polymer as a topical antimicrobial on the basis of the similarities of features to endogenous AMP and on the facts that NP108 is based on GRAS building blocks (GRAS notice 414) and is biodegradable without cytotoxicity and that ε-poly–l-lysines have a long history of safe and effective use as antimicrobials in the food industry (GRAS notices 135 and 336) (54). On the basis of the *in vitro* activity data and the potential ease of formulation determined in this study, NP108 will be taken further into preclinical/clinical development for the nasal decolonization of S. aureus and the prevention of SSI.

## MATERIALS AND METHODS

### Growth conditions and reagents.

All growth media and chemicals were purchased from Sigma-Aldrich (UK) unless otherwise stated. S. aureus DSMZ11729 (ATCC 33592) was obtained from the Deutsche Sammlung von Mikroorganismen und Zellkulturen GmbH (Germany). S. aureus ATCC 25923, S. aureus BAA-1717, S. epidermidis ATCC 35984, and S. epidermidis ATCC 12228 were obtained from the American Type Culture Collection (LGC Standards, UK). S. aureus NCTC10442, S. aureus NCTC10788 (ATCC 6538), and S. aureus NCTC6571 (ATCC 9144) were obtained from the National Collection of Type Cultures (Public Health England, UK). S. aureus SMRSA105, S. aureus SMRSA124, S. aureus SMRSA161, S. aureus EMRSA16, S. aureus MRSA8, S. aureus MRSA10, S. aureus MSSA1, S. aureus MSSA2, S. aureus MSSA6, and S. aureus MSSA8 were all isolated from dermal wounds and were generous gifts from Gail Ferguson and Isabel Cook of the Medical Microbiology Department, Aberdeen Royal Infirmary (UK). All other isolates were from the anterior nares of healthy volunteers.

### Isolation and characterization of nasal S. aureus.

Staphylococcus aureus was isolated from the noses of healthy volunteers using the protocol described in Investigation of nasal samples, a standard for microbiology investigations by the Public Health England, UK (55) after obtaining written informed consent. After their growth, colonies were assessed macroscopically on mannitol salt phenol red (MSPR) agar for growth and mannitol fermentation, and colonies were examined microscopically following Gram staining. Samples were further confirmed as S. aureus by the detection of coagulase activity and the presence of protein A in the cell wall (Staphylo Monotec test kit Plus; Sigma-Aldrich, UK). Samples were stored at −85°C in cation-adjusted Mueller-Hinton ([CA-MH] Mueller-Hinton broth 2; Sigma-Aldrich, UK) broth containing 30% (vol/vol) glycerol until required.

### Antimicrobial susceptibility testing.

The MICs of NP108 and antibiotics (vancomycin, fusidic acid [Discovery Fine Chemicals Ltd., UK], mupirocin [VWR, UK], and tobramycin [Discovery Fine Chemicals Ltd., UK]) were determined by the broth microdilution procedure (33). The MBCs of NP108 and antibiotics were measured following MIC determination using a 96-well replicator (Thermo Fisher Scientific). Briefly, after 20 h of incubation to determine the MICs, a 96-well replicator was used to transfer samples in triplicates from each well to 140-mm petri dishes containing CA-MH agar (no antimicrobials), which were incubated at 37°C until good growth was observed for positive (growth) control colonies (56). The MBC was determined as the lowest concentration of antimicrobial at which no growth was observed. Antimicrobial susceptibility testing was also carried out on S. aureus growing under low-nutrient conditions by the broth microdilution procedure (33), except that bacteria were grown in 0.1× CA-MH broth (one-tenth concentration) rather than normal concentration CA-MH broth.

### Generation of low-level mupirocin-resistant isolates.

Low-level mupirocin-resistant mutants (MIC_100_ = 8 to 64 mg/liter) were generated by exposing a 0.5 McFarland standard inoculum of mupirocin-sensitive isolates of S. aureus to doubling concentrations of mupirocin in CA-MH broth every 48 h for up to 16 d with incubation at 37°C, starting at a mupirocin concentration of 0.125 mg/liter on which all strains were able to grow. Mupirocin-resistant isolates were confirmed as S. aureus by growth on MSPR agar, macroscopic and microscopic appearance, the detection of coagulase activity, and the presence of protein A in the cell wall. Mupirocin MICs were determined by the broth microdilution procedure (33).

### Generation of small-colony variants.

Small-colony variants (SCV) of S. aureus ATCC 25923 (MSSA) and S. aureus DSMZ11279 (MRSA) were generated using the method described by Hoffman and coworkers (57) and characterized as described by Proctor and coworkers (40). SCV and controls were confirmed as S. aureus by growth on MSPR agar and their macroscopic and microscopic appearance. Antimicrobial susceptibility testing of SCV for NP018, mupirocin, and tobramycin was performed using the broth microdilution procedure (33). To maintain the SCV phenotype, 10 mg/liter 2-heptyl-4-hydroxyquinoline *N*-oxide (HQNO) was added to SCV cultures to prevent reversion to the wild-type phenotype, and SCV cultures were incubated until good growth was obtained in the untreated SCV controls (48 to 72 h) to compensate for the low growth rate of SCV.

### Generation of S. aureus biofilms and biofilm eradication.

Biofilms of S. aureus ATCC 25923 (MSSA) and S. aureus DSMZ11729 (MRSA) were generated using the microtiter plate biofilm assay procedure described by Cassat and coworkers (58) with the following modifications. The S. aureus culture was resuspended to the 0.5 McFarland standard in 0.1× tryptic soy broth ([TSB] 1:10 dilution of normal concentration TSB) supplemented with 0.05% (wt/vol) glucose and 0.3% (wt/vol) NaCl, and 200 μl was added to each well following the aspiration of plasma. Biofilms were established and treated in lower concentrations of growth medium to simulate the nutrient-poor conditions of the anterior nares (49). Plates were incubated statically at 37°C for 96 h to allow the biofilms to establish. For biofilm eradication studies, planktonic bacteria were removed from the wells after 96 h by gentle aspiration, and all wells were washed 3 times with sterile phosphate-buffered saline ([PBS] Thermo Fisher Scientific Ltd., UK). Antimicrobials (NP108 or mupirocin) dissolved in 0.1× TSB supplemented with 0.05% (wt/vol) glucose and 0.3% (wt/vol) NaCl were added to the relevant wells and incubated at 37°C for 24 h. Following treatment, biofilms were visualized by staining with crystal violet (59) to determine the amount of biofilm that had been eradicated relative to untreated control wells.

### Kinetics of NP108 bactericidal activity.

To determine the time that NP108 took to kill S. aureus, four isolates were assessed: S. aureus DSMZ11729 (MRSA), S. aureus ATCC 25923 (MSSA), S. aureus NCTC10442 (MRSA), and S. aureus NCTC10442 Mup^r^ (low-level mupirocin-resistant MRSA). S. aureus NCTC10442 Mup^r^ was generated at an earlier stage in this study (see above). For determination of the killing kinetics of exponentially growing S. aureus, cultures were diluted to the 0.5 McFarland standard, and 100 μl was inoculated into 10 ml CA-MH broth and grown overnight (16 to 18 h) at 37°C without shaking. For determination of the killing kinetics of stationary-phase S. aureus cultures, isolates were grown as described above but for 48 h. These time points had been previously determined to represent cultures in the exponential or stationary phase in preliminary experiments (data not shown). For exponentially growing cultures, samples of S. aureus were diluted to the 0.5 McFarland standard in CA-MH broth (prewarmed to 37°C) before exposure to NP108. For stationary-phase cultures, samples of S. aureus were diluted to the 0.5 McFarland standard in conditioned CA-MH broth (prewarmed to 37°C) before exposure to NP108. Conditioned CA-MH broth was prepared by growing the relevant isolate in CA-MH broth for 48 to 54 h, followed by the removal of cells by centrifugation (17,000 × *g* for 5 min), filter sterilization (0.22-μm polyethersulfone [PES] syringe filter; Jet Biofil), and storage at −20°C until required. Cultures of S. aureus were exposed to 4× MICs of NP108, and samples were removed hourly for 24 h. Samples were serially diluted in sterile PBS and plated on CA-MH agar plates and incubated for at least 24 h before colonies were enumerated.

### Determination of the effect of cationicity on antibacterial activity of NP108.

250 ml flasks of sterile 20 ml CA-MH broth were inoculated with an overnight culture of S. aureus SMRSA105 or S. aureus EMRSA16 (OD_625_ = 0.005) and incubated at 37°C with shaking (200 rpm) until OD_625_ = 0.1 to 0.3 (logarithmic growth). One half of the flasks were supplemented with PASA, a polyanionic compound used to neutralize the antimicrobial activity of cationic antimicrobial peptides (60), to a final concentration of 2.0% (wt/vol), immediately followed by the addition of 125 mg/liter NP108 (2× MIC_100_), 16 mg/liter vancomycin hydrochloride (2× MIC_100_), or 4 mg/liter fusidic acid, sodium salt (2× MIC_100_) (Discovery Fine Chemicals Ltd., UK) prepared in sterile CA-MH broth to the flasks with and without PASA. The cultures were incubated statically at 37°C, and at each specific time point (15, 30, and 60 min), a 0.02-ml aliquot was removed. Ten-fold serial dilutions to 10^−7^ were prepared in CA-MH broth, and each dilution was spread in triplicate onto CA-MH agar and incubated at 37°C until colonies were observable (∼24 h) in the untreated inoculated controls. Colonies were counted, and the percentage kill was determined based on the reduction in CFU (CFU/ml) in the treated samples relative to the untreated controls.

### Electron microscopy to determine the effect of NP108 on the cellular morphology of S. aureus.

Flasks (250 ml) containing 20 ml sterile CA-MH broth were inoculated with an overnight culture of S. aureus SMRSA105 (optical density at 625 nm [OD_625_] = 0.005) and incubated at 37°C with shaking (200 rpm) until an OD_625_ of 0.1 to 0.3 was achieved (logarithmic growth). Bacteria (500 μl from 10 ml for SEM) were added to an equal volume of CA-MH broth with 125 mg/liter NP108 (2× MIC_100_). NP108 and bacteria were incubated statically at 37°C for 30 min or 60 min followed by the addition of 2% (wt/vol) PASA and centrifugation (10 min at 13,000 × *g*). The cell pellets were resuspended and fixed in 1 ml 5% (wt/vol) glutaraldehyde in 0.2 M sodium phosphate buffer (pH 7.2) and were stored at 4°C. For SEM, cells were processed using a series of ethanol and hexamethyldisilazane dehydration procedures before gold mounting. For TEM, cells were dehydrated through a series of ethanol and acetone steps before being embedded in wax resin, stained with uranyl-acetate lead citrate to improve contrast, sectioned at 90 nm, and mounted onto copper grids. TEM and SEM micrographs were taken on a JEM-1400 Plus JEOL transmission electron microscope and a Zeiss EVO MA10 scanning electron microscope, respectively.

### Formulation of NP108 into aqueous hydrogels and antimicrobial susceptibility testing.

Sterile (autoclaved at 121°C for 20 min) water-soluble formulations containing NP108 were prepared. NP108 was aseptically added to all formulations at a concentration of 2.0% (wt/vol). Formulations without NP108 served as vehicle controls, and Bactroban nasal ointment (2.0% [wt/wt] mupirocin calcium) was used as an antibiotic kill control.

Antimicrobial susceptibility testing was carried out using S. aureus ATCC 25923 grown overnight on CA-MH agar plates at 37°C. The culture was diluted to the 0.5 McFarland standard in sterile PBS (∼10^8^ CFU/ml), spread plated on the surface of CA-MH plates made with 1.5% (wt/vol) ultra-high-resolution agarose (Melford Laboratories Ltd., UK) in place of agar to reduce electrostatic interactions that occur with the negatively charged sulfate and sugar components of the agaropectin in agar (61), and allowed to dry for 10 min at room temperature. Aliquots (0.2 ml) of formulations containing NP108 and formulation only controls were placed in the center of the lawn of S. aureus ATCC 25923 and incubated for 16 to 18 h at 37°C. Antimicrobial efficacy was demonstrated by a zone of no bacterial growth around the site of application of the formulation or Bactroban nasal ointment.

## Supplementary Material

Supplemental material
