# Supplementary material for: NP108, an Antimicrobial Polymer with Activity against Methicillin- and Mupirocin-Resistant Staphylococcus aureus
Source: Antimicrob Agents Chemother. 2017 Aug 24;61(9):e00502-17. doi: 10.1128/AAC.00502-17 (PMC5571353; doi:10.1128/AAC.00502-17)
Supplement: Supplemental material [file supp_61_9_e00502-17__index.html]

Supplemental material 

# NP108, an Antimicrobial Polymer with Activity against Methicillin- and Mupirocin-Resistant Staphylococcus aureus

## Supplemental material

- Supplemental file 1 -

  Supplemental Table S1 and Figure S1

  PDF, 207K
