# Supplementary material for: NP108, an Antimicrobial Polymer with Activity against Methicillin- and Mupirocin-Resistant Staphylococcus aureus
Source: Antimicrob Agents Chemother. 2017 Aug 24;61(9):e00502-17. doi: 10.1128/AAC.00502-17 (PMC5571353; doi:10.1128/AAC.00502-17)
Supplement: Supplemental material [file AAC.00502-17_zac009176448s1.pdf]

AAC00502-17 Supplemental Material

Table S1: In vitro antimicrobial activity of NP108 versus *Staphylococcus* spp. The minimum inhibitory concentration at which 100% growth was inhibited (MIC_{100}) and the minimum bactericidal concentration (MBC) at which all cells were killed (1) were determined by the broth microdilution procedure (2) and as described in (1), respectively. There were three technical replicates within each experiment and three biological replicates were carried out. MSSA – Methicillin-sensitive *S. aureus*, MRSA - Methicillin-resistant *S. aureus*, MSSE - Methicillin-sensitive *S. epidermidis*, GenR – Gentamicin resistant, SCV – Small colony variant. ND – Not determined.

Isolate	Features	Mean MIC_{100} (mg/L)	MBC (mg/L)
S. aureus DSMZ11729	MRSA, GenR	32	32
S. aureus NCTC10442	MRSA	32	32
S. aureus BAA-1717	MRSA	16	32
S. aureus EMRSA16	MRSA	20	20
S. aureus SMRSA105	MRSA	20	20
S. aureus SMRSA124	MRSA	20	20
S. aureus SMRSA161	MRSA	40	40
S. aureus MRSA8	MRSA	20	20
S. aureus ATCC25923	MSSA	32	32
S. aureus NS05R1	MSSA, Nasal isolate	16	32
S. aureus NCTC10788	MSSA	8	32
S. aureus NCTC6571	MSSA	32	32
S. aureus CB003	MSSA, Nasal isolate	16	ND
S. aureus CB004	MSSA, Nasal isolate	8	ND

S. aureus CB005	MSSA, Nasal isolate	500	ND
S. aureus CB006	MSSA, Nasal isolate	8	ND
S. aureus CB007	MSSA, Nasal isolate	250	ND
S. aureus CB008	MSSA, Nasal isolate	32	ND
S. aureus CB009	MSSA, Nasal isolate	64	ND
S. aureus FH-NAS-001	MSSA, Nasal isolate	16	ND
S. aureus FH-NAS-002	MSSA, Nasal isolate	32	ND
S. aureus FH-NAS-003	MSSA, Nasal isolate	16	ND
S. aureus FH-NAS-004	MSSA, Nasal isolate	16	ND
S. aureus MSSA1	MSSA	20	20
S. aureus MSSA2	MSSA	10	10
S. aureus MSSA6	MSSA	10	10
S. aureus MSSA8	MSSA	10	10
S. aureus MRSA10	MSSA	20	20
S. epidermidis ATCC35984	MSSE	8	8
S. epidermidis ATCC12228	MSSE	4	32
S. epidermidis V1A	MSSE, Nasal isolate	8	32

Figure S1: Antibacterial Efficacy of formulated NP108 versus *S. aureus* ATCC25923. A –Formulation A; B - Formulation A + 2.0% (w/v) NP108; C –Formulation B; D - Formulation B + 2.0% (w/v) NP108; E –Formulation C; F - Formulation C + 2.0% (w/v) NP108; G –Formulation D; H - Formulation D + 2.0% (w/v) NP108; I - Formulation E; J - Formulation E + 2.0% (w/v) NP108; K – Bactroban® (2.0% (w/w) mupirocin sodium). Plates were uniformly spread with exponential phase *S. aureus* ATCC25923 diluted to the 0.5 McFarland standard (~1 x 10^8 cfu/ml). Experiments were conducted in triplicate.

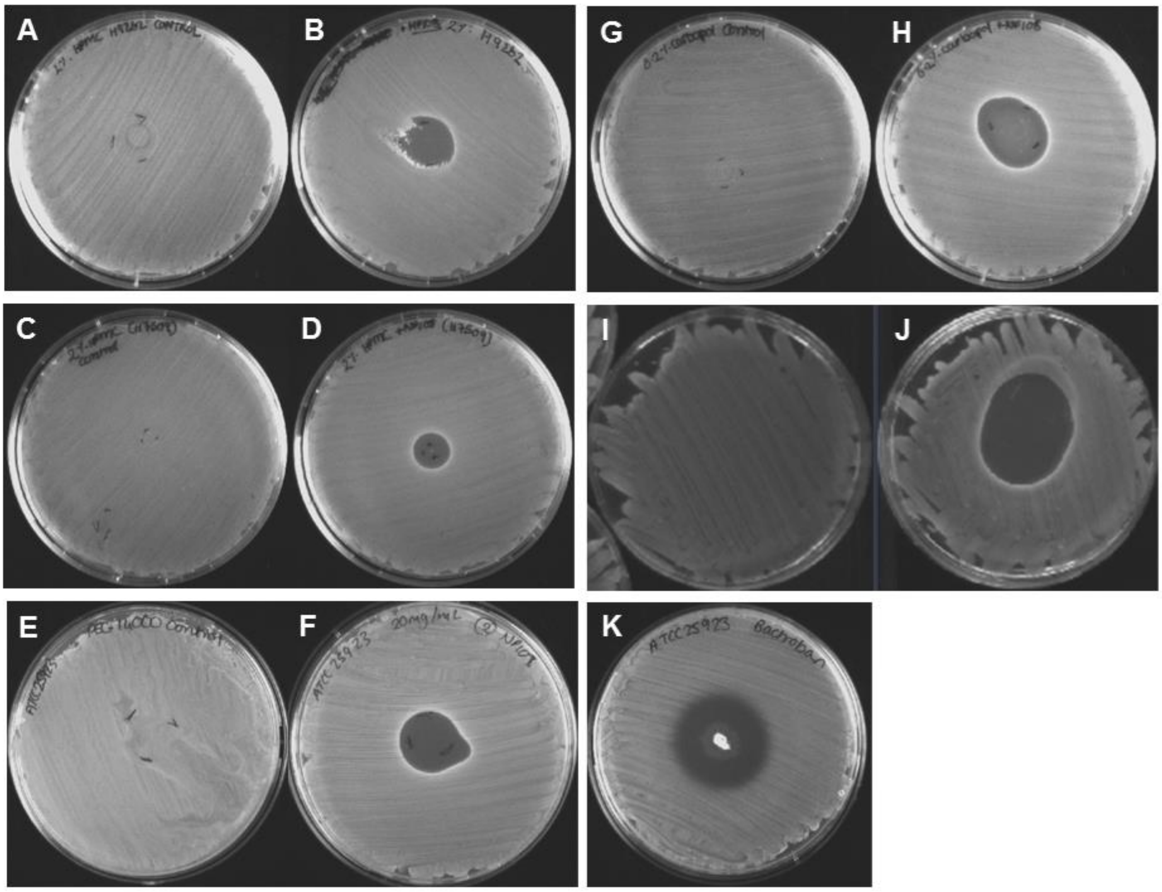

References

1. **O'Driscoll NH, Labovitiadi O, Cushnie TP, Matthews KH, Mercer DK, Lamb AJ.** 2013. Production and evaluation of an antimicrobial peptide-containing wafer formulation for topical application. Curr Microbiol **66:**271-278.

2. **CLSI.** 2015. Methods for dilution antimicrobial susceptibility tests for bacteria that grow aerobically; Approved Standard - Ninth Edition; M07-A10. Wayne, PA: Clinical and Laboratory Standards Institute.
